# Risk factors and survival of triple‐negative breast cancer among breast cancer patients: Ten‐year cross‐sectional study in the southwestern Iranian population

**DOI:** 10.1002/hsr2.1767

**Published:** 2023-12-17

**Authors:** Sholeh Arvandi, Sasan Razmjoo, Peyman Zaheri Abdevand

**Affiliations:** ^1^ Department of Radiotherapy, Faculty of Medicine, Golestan Hospital Ahvaz Jundishapur University of Medical Sciences Ahvaz Iran; ^2^ School of Medicine Ahvaz Jundishapur University of Medical Sciences Ahvaz Iran

**Keywords:** breast neoplasms, disease‐free survival, survival, triple negative breast neoplasms

## Abstract

**Background:**

Breast cancer results from genetic and epigenetic mutations, contributing significantly to cancer‐related morbidity and mortality. This study aimed to determine the prevalence and survival rates of triple‐negative breast cancer (TNBC) among breast cancer patients in southwestern Iran over a ten‐year period.

**Methods:**

This retrospective cross‐sectional study aims to assess prognostic factors associated with survival in women diagnosed with breast cancer in Iran's southwestern region over a ten‐year period (2007–2017). Data were collected from patients who visited the Clinical Oncology Department at Golestan Hospital in Ahvaz (the breast cancer center of the Southwestern country). The study enrolled women diagnosed with TNBC using a census method and data from medical records. The primary outcome (survival rates) and secondary outcomes (demographic data, diagnostic stages, and three receptors estrogen receptors [ER], progesterone receptor [PR], human epidermal growth factor receptor 2 [HER2] status) were collected.

**Results:**

Breast cancer was diagnosed in 2641 women over ten years; TNBC was diagnosed in 227 individuals (8.59%). Statistical analysis revealed a significant correlation between negative ER status and TNBC (*p* > 0.05). Furthermore, the prevalence of TNBC differed significantly from that of other types of breast cancer (*p* = 0.0001). The variables of age, HER2, PR, and TNBC grade did not differ significantly (*p* > 0.05). The overall disease‐free survival rate over 5 years was 88.1%, while the rate for individuals without recurrence was 77.97%.

**Conclusion:**

This study highlights a differentially low incidence of TNBC in the southwestern part of Iran when compared to other regions; genetic or epigenetic influences may explain this discrepancy. ER‐negative status is a crucial prognostic indicator in diagnosing TNBC. The incidence of this disease is expected to rise by 100% in 2 years and 77.97% in 5 years.

## 1 INTRODUCTION

Current knowledge indicates that breast cancer (BC) comprises numerous subgroups, each distinguished by a unique pathophysiology, treatment outcomes, and response to particular therapies.^1^, ^2^ This malignancy is distinguished by the lack of expression of progesterone receptors (PR) and estrogen receptors (ER), in addition to an increased level of human epidermal growth factor receptor 2 (HER2) receptors.^3^ As a result, this condition is classified into distinct molecular subgroups. One of the variants that differentiates triple‐negative breast cancer (TNBC) is its aggressive nature. TNBC is characterized by the absence of HER2 receptors, which are targeted by trastuzumab, and ER and PR receptors that are suitable for hormonal intervention.

Consequently, targeted therapies for this group of BC patients are rendered less effective.^4^, ^5^ In contrast, TNBC comprises a proportionately high 12%–17% of all BCs and frequently manifests a recurring pattern.^6^ The current average survival rate for this disease is around 10 months, with a 5‐year survival rate of 65% for localized tumors and 11% for cases with distant organ metastasis.^7^

Examining the genomic phenotype of epithelial cells, notwithstanding the considerable expenses involved, represents a feasible strategy to tackle the challenge above. When determining the subtype of cancer in a patient, immunohistochemistry provides a more practical and cost‐effective method.^8^ Estrogen receptor (ER), progesterone receptor (PR), and HER2 status evaluations are components of conventional pathological and immunohistochemical analyses.^9^ A triple‐negative tumor is one in which all three of these markers produce negative results.^10^ Triple‐negative breast carcinomas are generally observed in women below the age of 50 and are distinguished by their aggressive nature and unfavorable prognosis. In comparison to other molecular subtypes, they exhibit heightened aggression despite demonstrating a robust initial response to chemotherapy.^4^, ^11^ Despite the lack of a standard treatment regimen for TNBC and the scarcity of available data, researchers have endeavored to identify molecular pathways critical to tumor proliferation.^12^, ^13^ The discovery of these mechanisms raises hopes for the development of targeted therapeutic strategies. Given the high prevalence of BC and the importance of distinguishing TNBC patients from those with nontriple‐negative tumors due to different treatment protocols, a thorough investigation of TNBC epidemiology is critical. Furthermore, due to a shortage of studies addressing this topic in Iran's southwestern region, this study was undertaken to determine the relative prevalence and survival rates of TNBC patients in this region over a decade among breast cancer patients.

## 2 MATERIAL AND METHODS

This study adhered to the Strengthening the Reporting of Observational Studies in Epidemiology guidelines for cross‐sectional studies.^14^

### 2.1 Study design and setting

A retrospective cross‐sectional study was conducted to explore prognostic factors linked to the survival of women diagnosed with TNBC within the southwestern region of Iran over a defined period. Solely TNBC cases were encompassed in this investigation. The study sought to gauge the impact of TNBC in the southwestern Iranian context spanning from 2007 to 2017. Ethical clearance for this study was secured from the ethics committee of Jundishapur University of Medical Sciences (AJUMS), Ahvaz (IR. AJUMS. REC.1397.029). Moreover, all patient data obtained were handled with strict confidentiality.

### 2.2 Participants

#### 2.2.1 Inclusion and exclusion criteria

Female patients diagnosed with TNBC, who had sought medical care at the Clinical Oncology Department of Golestan Hospital in Ahvaz (a prominent breast cancer center in the southwestern region of the nation), were incorporated into this study. In instances where patient records displayed gaps, concerted efforts were made to supplement the lacking information through telephonic interactions with the patients. Individuals unresponsive to these calls or those lacking requisite information were excluded from the study. Patients presenting undefined status for any of the following receptors ‐ HER2, ER, and PR ‐ or having incomplete follow‐up data were also omitted.

### 2.3 Data collection

Patient data, diagnosed with TNBC and attending the Clinical Oncology Department at Golestan Hospital in Ahvaz, were amassed through a census approach, drawing from available medical records. The patients' general diagnostic stage was categorized in alignment with the American Joint Committee on Cancer seventh edition staging classification. Parameters encompassing tumor size, lymph node status, and metastasis were meticulously collated. Each patient furnished demographic data and pertinent research variables via a questionnaire. These essential variables were culled from patient files, occasionally through telephone inquiries, and subsequently transcribed into the questionnaire. Patients were grouped into diagnostic stages ranging from 1 to 3. HER2 IHC status was classified as HER2‐negative or HER2‐positive, following the American Society of Clinical Oncology guidelines. Untested status was disregarded, and analysis excluded missing data employing the “unknown” code. Hormone receptor status, a fusion of ER and PR, was denoted as either positive or negative. Patients lacking all three receptors (ER, PR, HER2) were identified as having TNBC, while those with at least one of these receptors were categorized as non‐TNBC. The overall survival spanned from the time of diagnosis or commencement of treatment to the last follow‐up or the patient's demise. Disease‐free survival was delineated from the point of diagnosis or initiation of treatment until confirmed disease recurrence based on imaging or biopsy. Age at diagnosis was stratified into six groups: under 35 years (group 1), 35–45 years (group 2), 45–55 years (group 3), 55–65 years (group 4), 65–75 years (group 5), and over 75 years (group 6).

### 2.4 Statistical methods

All collected data underwent analysis utilizing SPSS version 22 software, adopting a significance threshold of 0.05. Descriptive statistics were presented as mean (±standard deviation), alongside frequency distribution tables and percentages. Inferential statistics comprised independent t‐tests and Chi‐square tests. The Kaplan‐Meier method was employed to compute patients' survival rates. The influence of auxiliary variables, such as age at diagnosis, on patients' survival duration was evaluated via the Log‐Rank Mantel‐Cox test.

## 3 RESULTS

### 3.1 Participant overview

Across a span of ten years, 2641 women received diagnoses of breast cancer. As per the findings, the highest proportion of patients (20.71%) fell within the age bracket of 35 to 45 years, while the lowest proportion (2.12%) was recorded among individuals aged above 75 years. The mean age of the patients was documented as 48.12 ± 12.7 years. Additional demographic particulars are delineated in Table 1.

**Table 1:** Demographics and characteristics of women diagnosed with TNBC in southern west of Iran.

Overall		No.	%	*p* value
**Age**	**<35**	425	16.09	0.31
	**35–45**	547	20.71	
	**45–55**	445	16.85	
	**55–65**	332	12.57	
	**65–75**	222	8.4	
	**>75**	56	2.12	
**ER**		960	36.35	**0.007**
**PR**		1146	43.39	0.45
**HER2**		1532	58	0.91
**ER‐/PR‐/HER2‐(TNBC)**		227	8.59	**<0.0001**
**TNBC grade**	**1**	16	7.04	0.69
	**2**	63	27.76	
	**3**	148	65.2	

Abbreviations: ER, estrogen receptors; HER2, human epidermal growth factor receptor 2; PR, progesterone receptors; TNBC, triple‐negativebreast cancer.

### 3.2 Prognostic indicators

Among the cohort, 245 individuals (9.27%) were identified as having TNBC, and after accounting for incomplete records, a total of 227 subjects were encompassed in the study (Table 1). Rigorous statistical scrutiny exposed a noteworthy variance in ER‐negative status across the patient pool (*p* = 0.007). The prevalence of individuals afflicted with TNBC diverged significantly from other patients (*p* < 0.0001). However, variables encompassing age, PR, HER2, and TNBC grade did not manifest statistically substantial disparities (*p* > 0.05).

### 3.3 Survival rates

**Figure 1:**
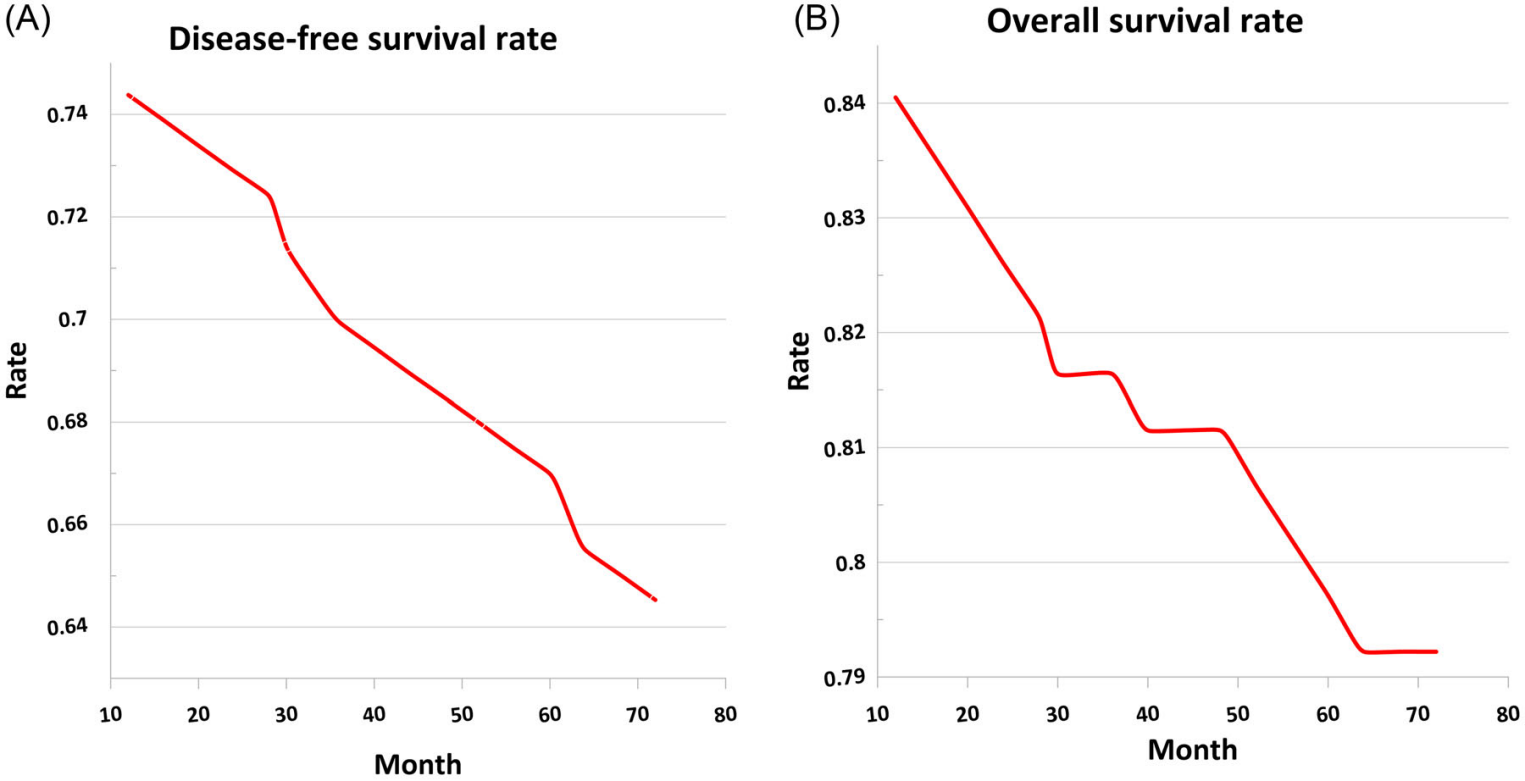
(A) Two‐ and 5‐year disease‐free survival rate of triple negative breast cancer. (B) Two‐ and 5‐year overall survival rate of triple negative breast cancer.

The 5‐year overall survival rate stood at 88.1%, relative to 77.97% for patients who did not experience recurrence. When examining the influence of age at diagnosis on the 2‐year survival rate, the Log‐Rank statistic yielded 0.230, while for the 5‐year survival rate, it amounted to 0.102 (Table 2 and Figure 1). For patients with TNBC, the 2‐year disease‐free survival rate was noted as 82.0%, extending to 78.0% for the 5‐year duration (Table 2 and Figure 1). The comprehensive analysis of the 2‐year overall survival rate, in conjunction with age at diagnosis, unveiled a Log‐Rank statistic of 0.140. Parallelly, the 5‐year overall survival rate displayed a statistic of 0.092. Delving deeper, the 2‐year overall survival rate among TNBC patients reached 89.0%, while the corresponding 5‐year statistic rested at 82.0% (Table 2 and Figure 1). Notably, the significant levels for both overall survival and disease‐free survival concerning the age variable did not present discernible meaningful distinctions.

**Table 2:** Two‐ and five‐year disease‐free survival based on age at diagnosis in patients with triple‐negative breast cancer.

		Disease‐free survival		Overall survival	
		2 years	5 years	2 years	5 years
**Age of disease diagnosis, *N* (%)**	**≤ 50 years**	(55.07) 125	(60.45) 107	(54.19) 123	(45.5) 91
	**>50 years**	(44.93) 102	(39.54) 70	(45.81) 104	(54.5) 109
**Total, *N* (%)**		227 (100)	177 (77.97)	227 (100)	200 (88.1)
**Log‐rank test**		0.23	0.102	0.14	0.092

## 4 DISCUSSION

In this study, the estimation of the disease‐free and overall survivals and the factors affecting it after the treatment of TNBC patients referred to a prominent breast cancer center in the southwestern region of the nation in Iran, which is the reference hospital of social security for the treatment of cancers, was investigated. In the context of this retrospective study, spanning a decade, examination of prognostic elements and patient survival rates unveiled a percentage of 8.59 for breast cancer cases treated therapeutically during the past 10 years to possess TNBC status, indicative of TNBC. Other studies have reported TNBC prevalence rates among women varying from 8% to 29.8%.^12^, ^15^, ^16^, ^17^, ^18^, ^19^, ^20^, ^21^, ^22^, ^23^, ^24^, ^25^, ^26^ Research reveals an anticipated annual incidence of 2.3 million fresh cases of BC, accounting for around 11.7% of all cancer occurrences.^27^ A significant obstacle confronted by individuals with TNBC lies in the limitation of prescribing hormonal and targeted therapies.^13^, ^28^ Hence, identifying clinical, pathological, and prognostic advancements and orchestrating therapeutic strategies for this group assume paramount significance.^28^ Nearly 80% of breast cancer cases, including TNBC, are observed in women aged over 50. The vulnerability to TNBC surges from 1.5% at age 40 to over 4% in those aged 70 and above.^27^, ^29^ Furthermore, TNBC tends to be more prevalent in women under 40, with an incidence rate of 4.2%.^20^, ^30^

Furthermore, a meta‐analysis study underscored a TNBC prevalence of 27% across Africa, with notable variations within the continent. Notably, Western African nations exhibited a substantial gap, with a prevalence of 45.7% compared to 14.9% in the central region.^11^ Similarly, investigations carried out in India yielded comparable prevalence rates to those in Africa, citing 27% and 25.04%.^31^, ^32^ In contrast, TNBC prevalence in the United States stood at 13.7%.^33^ Interestingly, the findings of this study converge with the global TNBC prevalence, revealing a lower frequency in comparison to the mentioned countries. Nevertheless, juxtaposed against other research conducted in Iran, this study's outcomes imply a diminished TNBC prevalence in the southwestern region.^18^, ^19^, ^26^, ^34^

The outcomes of this study delineate that 63.65%, 56.61%, and 42% of patients exhibited positive results for estrogen, progesterone, and HER2 receptors, respectively. However, solely the estrogen receptor status displayed statistical significance among the patients. In a parallel vein, Ghosh et al. (2008) documented a 2.51% positivity rate for estrogen and PRs,^15^ collectively underscoring a congruence between the present study's findings and results from diverse regions. Most participants in this study were categorized as Grade 3 TNBC (65.2%), with no significant statistical divergence noted amidst grading categories. This phenomenon is elucidated in light of the observations made by Xiao et al., spotlighting a robust connection between elevated disease grade and TNBC diagnosis, serving as a prognostic marker.^35^ Furthermore, studies have consistently demonstrated a positive correlation between clinical stage and pathological grade, establishing an autonomous risk factor for TNBC patients.^36^, ^37^, ^38^

From this study, it is evident that survival rates among women with TNBC do not exhibit statistically significant variation about age. The assessment of overall survival and disease‐free survival was conducted independently for age groups below 50 and those above 50 years. However, these statistics failed to yield statistically significant disparities within the categorized cohorts (*p* > 0.05). Notably, the 5‐year overall survival and disease‐free survival rates stood at 88.1% and 77.97%, respectively. Similarly, in a separate study, the 5‐year overall survival and disease‐free survival rates were recorded as 74% and 80%, correspondingly.^39^ Another investigation revealed that disease‐free survival among breast cancer patients with positive hormone receptors was notably abbreviated, evidenced by a 5‐year survival rate of 80%.^15^ In line with this, Caradela (2005) documented a 3‐year survival rate of 82.7% among these subjects in an Italian study.^24^ Furthermore, congruent with the outcomes of the present study, another inquiry demonstrated 5‐year overall survival rates of 81.28% and 86.50% for TNBC and non‐TNBC, respectively,^40^ and overall survival 82% for west Iranian's patients.^41^

Ultimately, it is discerned that TNBC manifests an inherently aggressive nature, with constrained treatment avenues and a grimmer prognosis. Consequently, the identification and acknowledgment of biomarkers assume paramount significance, underscoring their potential contribution to efficacious screening. Nevertheless, this study contends with certain limitations, encompassing the paucity of data stemming from both the southwestern region of Iran and the nation at large. Furthermore, the unavailability of specific patient‐related data, encompassing racial and ethnic disparities, curtails an in‐depth discussion regarding the potential influence of these factors on disease prognosis. Moreover, health‐related data, insurance status, lifestyle facets (including body mass index, weight, physical activity, and diet), breast density, and genetic testing remain unaccounted for, despite their capacity to impact breast cancer outcomes.

## 5 CONCLUSIONS

The conclusions drawn from the present study illuminate nuanced distinctions in risk factors existing within distinct subgroups of breast cancer. ER‐negative status is a vital prognostic factor for diagnosing TNBC. The incidence of this disease is expected to rise by 100% in 2 years and 77.97% in 5 years. Noteworthy is the observation that the prevalence of TNBC within the southwestern Iranian region registers a lower frequency compared to other locales, a disparity that could potentially stem from genetic or epigenetic influences. These findings underscore the imperative to explore novel risk determinants such as genetics, epigenetics, biomarkers, and environmental exposures, all critical in comprehending the intricacies of risk associated with distinct subcategories of TNBC within this specific region. Furthermore, emphasizing the pivotal role of early detection in curbing mortality rates, the study underscores the urgency of characterizing patients afflicted with this form of cancer, thereby presenting prospective solutions and essential insights aimed at ameliorating the incidence of this malignancy while mitigating potential risks and challenges faced by patients.

## AUTHOR CONTRIBUTIONS

**Sholeh Arvandi**: Conceptualization; data curation; formal analysis; investigation; methodology; project administration; software; supervision; writing—original draft; writing—review and editing. **Sasan Razmjoo**: Resources; writing—original draft. **Peyman Zaheri Abdevand**: Resources; writing—original draft; writing—review and editing.

## CONFLICT OF INTEREST STATEMENT

The authors declare no conflict of interest.

## ETHICS STATEMENT

The initial proposal of the work was approved by the Institutional Review Board (IRB) and Ethics Committee of the AJUMS Iran (code: IR. AJUMS. REC.1397.029). This study used recorded data of patients and no intervention was done on included population.

## TRANSPARENCY STATEMENT

The lead author Sholeh Arvandi affirms that this manuscript is an honest, accurate, and transparent account of the study being reported; that no important aspects of the study have been omitted; and that any discrepancies from the study as planned (and, if relevant, registered) have been explained.

## Data Availability

Data available on request from the authors.
